# Supplementary material for: Incidence and Predictors for Oncologic Etiologies in Chinese Children with Pituitary Stalk Thickening
Source: Cancers (Basel). 2023 Aug 2;15(15):3935. doi: 10.3390/cancers15153935 (PMC10417368; doi:10.3390/cancers15153935)
Supplement: Supplementary file 1 [file cancers-15-03935-s001.zip › cancers-2492582-supplementary.pdf]

Supplementary Table S1. Baseline Demographics of non-neoplastic versus neoplastic groups.

		**Idiopathic (n=14)**	**Neoplastic causes (n=14)**	**Total**
**Sex**	Female	12 (85.7%)	6 (42.9%)	18
	Male	2 (14.3%)	8 (57.1%)	10
**Age at first symptoms (median and range in years)**		9.3 (3.6–16)	11 (5–16.3)	9.7 (3.6–16.3)
**Age at diagnosis of PST (median and range in years)**		10.7 (3.8–16.2)	11.1 (5.2–16.5)	10.9 (3.8–16.5)
**Presenting Symptoms**	Polyuria/polydipsia	4	13	17
	Growth retardation	2	7	9
	Precocious puberty	7	1	8
	Delayed puberty	2	3	5
	Headache	2	3	5
	Visual problems	4	5	9
	Weight loss	1	2	3
	Lethargy	1	2	3
	Dizziness	0	1	1
	Poor appetite	2	1	3
	Decrease libido	0	1	1
**Visual Problems**	Visual field	1	1	2
	Visual acuity	0	0	0
	Papilledema	0	1	1
	Extropia/diplopia	2	2	4
	Parinaud syndrome	0	1	1
	Raised intra-ocular pressure	0	1	1
	Amblyopia	1	0	1
**Biopsy performed**	No	14 (100%)	3 (21.4%)	17
	Yes	0 (0%)	11 (78.6%)	11
**Stalk thickeness at initial MRI**	Mild (<4mm)	11 (78.6%)	4 (28.6%)	15
	Moderate (4mm-<6.5mm)	3 (21.4%)	5 (35.7%)	8
	Severe (≥6.5mm)	0 (0%)	5 (35.7%)	5
**Maximal stalk thickness during FU**	<6.5mm	14 (100%)	2 (14.3 %)	16
	≥6.5mm	0 (0%)	12 (85.7 %)	12
**Outcome**	Alive	14	13	27
	Died	0	1	1
